# Recombinant human thyrotropin versus thyroid hormone withdrawal preparation for radioiodine ablation in differentiated thyroid cancer: Experience in a South Taiwanese medical center

**DOI:** 10.1002/kjm2.12621

**Published:** 2022-11-30

**Authors:** Jia‐Ruei Tsai, Shu‐Ting Wu, Shun‐Yu Chi, Yi‐Ting Yang, Yi‐Chia Chan, Lay San Lim, Yvonne Ee Wern Chiew, Wen‐Chieh Chen, Yung‐Nien Chen, Chen‐Kai Chou

**Affiliations:** ^1^ Division of Endocrinology and Metabolism, Department of Internal Medicine Kaohsiung Chang Gung Memorial Hospital and Chang Gung University College of Medicine Kaohsiung City Taiwan; ^2^ Division of General Surgery, Department of Surgery Kaohsiung Chang Gung Memorial Hospital and Chang Gung University College of Medicine Kaohsiung City Taiwan

**Keywords:** differentiated thyroid cancer, excellent response rate, radioiodine ablation, recombinant human thyrotropin, thyroid hormone withdrawal

## Abstract

This retrospective study was designed to compare the treatment response of patients with differentiated thyroid cancer (DTC) prepared for radioiodine ablation (RIA) with thyroid hormone withdrawal (THW) or recombinant human thyrotropin (rhTSH) stimulation. Patients with DTC were followed‐up retrospectively between 2013 and 2018 in Kaohsiung Chang Gung Memorial Hospital, Taiwan. We compared the excellent response ratios between THW (49.9%) and rhTSH (50.1%) stimulation. Patients were then divided into subgroups, on the basis of age, sex, extrathyroidal extension, lymph node metastasis, and tumor‐node‐metastasis stage, for analysis. In all, 647 patients were followed‐up after RIA. The ratios of THW or rhTSH use in the different subgroups were not statistically significant. In all the patients, the excellent response rate with THW and rhTSH was 80% and 76.5%, respectively, which was not statistically significant. The subgroup analysis, including age, sex, extrathyroidal extension, lymph node metastasis, and tumor‐node‐metastasis stage (low and high risk), showed similar results. Furthermore, the *logistic* regression analysis revealed no statistically significant differences among the subgroups. The multivariate analysis showed extrathyroidal extension, lymph node metastasis, and high I^131^ dose were the prognostic factors affecting the excellent response rate. In conclusion, the THW and rhTSH preparations for RIA were similar in terms of the excellent response rates and subgroup clinical outcomes.

## 1 INTRODUCTION

Although the global incidence of thyroid cancer has been continuously increasing in the past decades, mortality rates have been decreasing gradually, such as the rate in Asia.^1^ This increase in incidence has been associated with the development of diagnostic and treatment strategies. However, there has been a consequent increase in the global economic burden as well.^2^, ^3^ Among the types of thyroid cancer, differentiated thyroid cancer (DTC) accounts for more than 90% of all thyroid malignancies and can be subdivided into papillary thyroid cancer (PTC, 85%–90%), follicular thyroid cancer (FTC; 5%–10%), and Hürthle (oncocytic) cell thyroid cancer (2%–5%).^4^, ^5^

The primary therapy for PTC is surgery,^6^ and radioiodine ablation (RIA) is arranged for consecutive treatment except extremely small DTC (≤1 cm) with no extrathyroidal extension, no lymph node (LN) involvement, and no distant metastases. The 2015 American Thyroid Association Guidelines suggest that patients with intermediate or high risk of recurrence of DTC receive RIA after total thyroidectomy as the standard of care^6^, ^7^ as this has been associated with better outcomes.^8^, ^9^

There are two different preparation strategies before RIA, namely thyroid hormone withdrawal (THW) and recombinant human thyrotropin preparation (rhTSH). If THW is planned before RIA, thyroxine should be withdrawn for 4 weeks with a target thyroid stimulating hormone (TSH) level of >30 mIU/L.^9^, ^10^, ^11^ Furthermore, rhTSH before RIA is an alternative to avoid hypothyroidism and has been associated with an improved quality of life as compared to THW.^12^, ^13^, ^14^ In addition to the relatively short‐lived increase in serum TSH levels, rhTSH administration can reduce the risk of tumor growth compressive symptoms in patients with a history of rapid progression during a protracted period of TSH elevation.^15^ Furthermore, patients who receive rhTSH preparation may have lower mean residence times of radioiodine in the body than those who receive THW preparation.^16^

Dynamic risk assessment has been suggested to emphasize the response to therapy for each patient on every follow‐up visit using diagnostic tests, including thyroglobulin (Tg) levels, thyroglobulin antibodies, and thyroid ultrasonography.^9^, ^17^, ^18^, ^19^ In fact, response to therapy according to the dynamic risk assessment can predict outcomes over a 10‐year period by using the data from the first 2 years of follow‐up alone, allowing physicians to adjust their long‐term strategies.^17^, ^20^

Ujjal et al. reported in their *randomized controlled trial* on low and high dose RIA under rhTSH or THW on DTC that the ablation success rate is similar between rhTSH and THW preparation.^21^ For intermediate‐ and high‐risk patients without any known distant metastasis, the rhTSH preparation for RIA can be effectively used.^22^ Jian et al. conducted a meta‐analysis that demonstrated similar ablation rates between rhTSH and THW for patients with DTC.^23^ Moreover, in metastatic DTC, rhTSH preparation can be used to achieve the benefits of RIA that are comparable with those of THW preparation.^24^

To the best of our knowledge, there has yet to be a comparison of the response to therapy outcomes and the subgroup analysis between the use of rhTSH and that of THW for low‐ to high‐risk DTC patients in Taiwan. Therefore, this study aimed to investigate the treatment response differences between rhTSH and THW as pre‐RIA protocol preparation by using a subgroup analysis.

## 2 METHODS

### 2.1 Study design and patient recruitment

The current study is a single medical center, retrospective, cohort study design in a South Taiwanese medical center. The primary objective was to demonstrate whether rhTSH and THW affected the treatment response in patients with DTC and analyze the clinical outcomes in subgroups. We retrospectively reviewed the medical records of 647 patients. All the patients were >18 years of age at the time of total thyroidectomy and radioactive iodine for DTC in Kaohsiung Chang Gung Memorial Hospital between 2013 and 2018. DTC staging was based on the American Joint Committee on Cancer Tumor‐Node‐Metastasis (AJCC‐TNM) Classification, 7th edition (2009), for DTC.^25^

**FIGURE 1:**
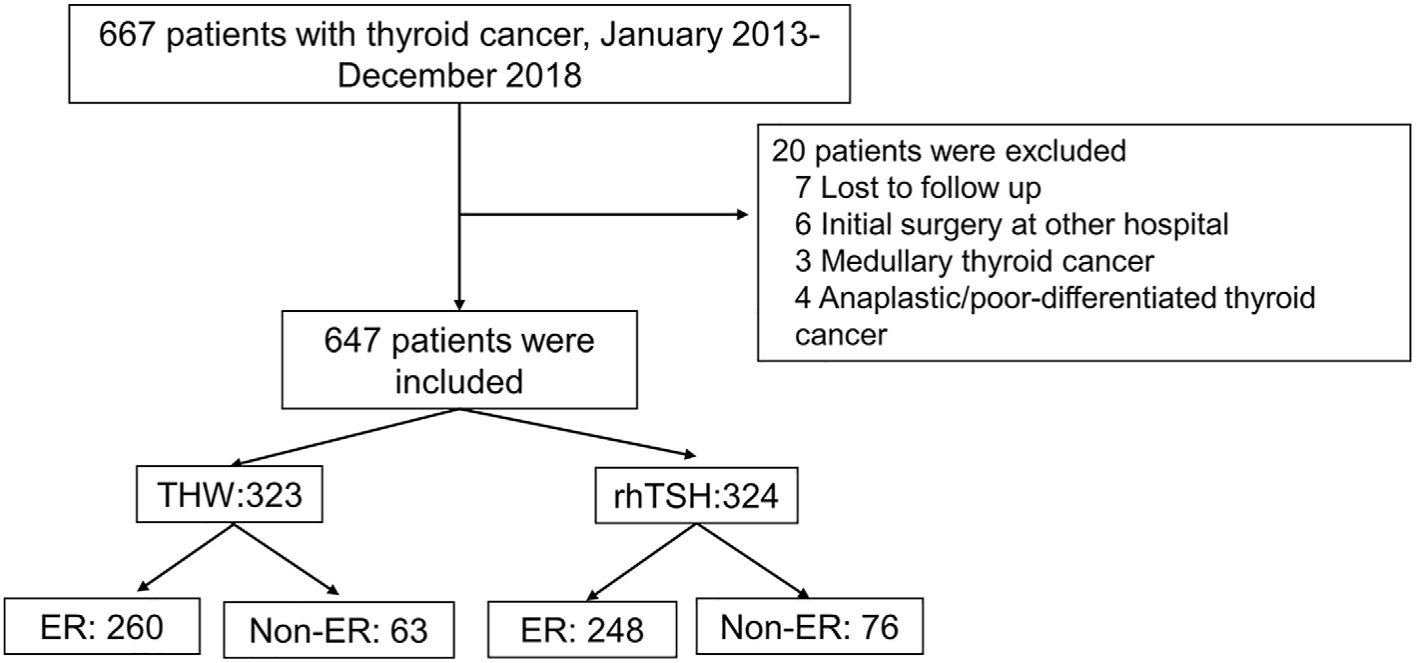
Algorithm for subject enrollment. ER, excellent response; Non‐ER, non‐excellent response; rhTSH, recombinant human thyrotropin; TWH, thyroid hormone withdrawal.

Patients had to be histologically confirmed to have DTC and followed‐up for at least 1 year to be included in the study. Patients with poorly differentiated, anaplastic, or medullary carcinoma; those with previous treatment for thyroid cancer other than surgery; and those lost to follow‐up were excluded (Figure 1). This study was approved by the Institutional Review Board of Chang Gung Memorial Hospital (IRB No.: 201801481B0C501).

### 2.2 Treatment protocol

Patients who were highly suspected to have thyroid cancer were referred to a general surgeon for total thyroidectomy. After undergoing total thyroidectomy with or without neck dissection, the patients underwent evaluations with endocrinologists, general surgeons, and nuclear radiologists. All the patients were staged as T1–T3, N0–N3, and M0–M1, according to the AJCC Thyroid Cancer Staging, 7th edition. If the tumor size was >1 cm or the patient was deemed intermediate−/high‐risk based on the 2015 American Thyroid Association Guidelines, RIA with I^131^ was recommended (30–200 mCi, dose selected according to disease severity).^9^, ^26^ The patients received a low‐iodine diet for at least 4 weeks prior to RIA, which was arranged approximately 6 months following the total thyroidectomy. Prior to ablation, conventional THW or rhTSH injection was selected on the basis of Taiwan's national healthcare program and the patients' comorbidities. The patients could choose THW or rhTSH after shared decision making with the physicians.

If the patients selected THW, thyroxine was withheld at least 4 weeks prior to RIA to reach a TSH level ≥ 30 mU/L and I^131^ was ingested. The other group of patients who chose rhTSH, were injected 0.9‐mg rhTSH intramuscularly, followed 24 h later by a second 0.9‐mg dose; I^131^ was ingested within 24 h of the second injection. Currently, rhTSH is not routinely included in Taiwan's national healthcare program except in the cases of severe hypothyroidism, multiple comorbidities, and thyroid cancer recurrence.

### 2.3 Outcome assessment

Following RIA, we evaluated the response to therapy for at least 1 year, following the 2015 ATA Guidelines.^9^ One to 2 years after RIA, serum non‐stimulated Tg and antithyroglobulin antibody (TgAb) levels were obtained, and ultrasonography and diagnostic whole‐body scan (DxWBS) were performed for imaging. Excellent treatment response was defined as having non‐stimulated Tg levels of <0.2 ng/ml, undetectable TgAb, and negative imaging on neck ultrasonography and DxWBS.^9^ Biological incomplete response was defined as having abnormal Tg levels, rising anti‐Tg antibody levels, and lack of localizable disease on imaging. Structural incomplete response was defined when persistent, newly loco‐regional, or distant metastases were revealed on thyroid ultrasonography or other imaging.^9^, ^27^, ^28^ Lastly, indeterminate response was defined as having non‐specific biochemical or structural findings, which could not be classified as either benign or malignant.^9^

Patients were divided into two groups (excellent response and non‐excellent response) for analysis on the basis of the results of the response to therapy.

The non‐excellent response group included biochemical incomplete response, structural incomplete response, and indeterminate response.

### 2.4 Statistical analysis

Statistical analyses were performed using the SPSS, version 24.0 (IBM). Descriptive statistics were presented as numbers and percentages, means ± standard error, and medians and ranges. Pearson's chi‐squared test was used to determine statistically significant differences between rhTSH and THW and the ratio of excellent response in the subgroup analysis. Clinical factors that might affect the rate of excellent response were assessed using univariate and multivariate logistic regression analyses. Statistical significance was set at a *p*‐value of <0.05.

## 3 RESULTS

We identified 647 DTC patients that met the study criteria during the study period from January 2013 to December 2018. The clinical features of these patients are reported in Table 1.

**TABLE 1:** Characteristics of patients between 2013 and 2018*

Clinical characteristics	No. (%)	Thyroid hormone withdrawal	rhTSH	*p*
Total number of patients	647	323 (50.1%)	324 (49.9%)	
Age, years	49.48 ± 13.74	49.29 ± 13.06	49.66 ± 14.40	0.081
Female	487 (75.3%)	235 (72.8%)	252 (77.8%)	0.139
Tumor type				
Papillary carcinoma	613 (94.7%)	306 (94.7%)	307 (94.8%)	0.952
Hürthle cell carcinoma	7 (1.1%)	5 (1.6%)	2 (0.6%)	
Follicular carcinoma	27 (4.1%)	12 (3.7%)	15 (4.6%)	
TNM stage (AJCC‐7th edition)				
Low risk (stage I + II)	484 (74.8%)	246 (76.2%)	238 (73.5%)	0.428
High risk (stage III + IV)	163 (25.2%)	77 (23.8%)	86 (26.5%)	
Extrathyroidal extension	276 (42.7%)	127 (39.3%)	149 (46.0%)	0.086
Lymph node metastasis	261 (40.3%)	122 (37.8%)	139 (42.9%)	0.184
Distant metastasis	17 (2.6%)	6 (1.9%)	11 (3.4%)	0.22
I^131^ dose (mCi)	96.36 ± 34.99	95.44 ± 33.02	97.35 ± 36.9	0.081
Excellent response	508 (78.5%)	260 (80.5%)	248 (76.5%)	0.221

*Note*: Data are presented as n (%) or mean ± standard deviation. rhTSH, recombinant human thyrotropin.

Abbreviations: AJCC, American Joint Cancer Committee; TNM, tumor‐node‐metastasis.

In the study, the mean age of all the patients was 49.48 ± 13.74 years, and the proportion of females was 75.3%. The ratio of papillary carcinoma, follicular carcinoma, and Hürthle cell carcinoma was 94.7%, 4.1%, and 1.1%. This study staged patients according to AJCC Thyroid Cancer Staging, 7th edition (2009), and classified 388 of the patients (60.0%) as stage I, 96 (14.8%) as stage II, 116 (17.9%) as stage III, and 47 (7.3%) as stage IV. The number of patients with LN metastasis and extrathyroidal extension was 261 (40.3%) and 276 (42.7%), respectively, and 17 patients (2.6%) had distant metastasis. Of these patients, 324 (50.1%) patients received rhTSH injection for RIA, and 508 [78.5%] patients had excellent response (Table 1).

### 3.1 Subgroup comparisons

Patients were divided into two groups according to the method of pre‐RIA preparation. We divided the patients with stages I–II as the low‐risk group and patients with stages III–IV as the high‐risk group.^28^

The ratios of THW versus rhTSH in terms of age, sex, TNM staging (low or high risk), extrathyroidal extension, LN metastasis, and excellent response rate were not significantly different (Table 1). The rate of excellent response in THW and rhTSH for the pre‐RIA preparation was 80% and 76.5%, respectively, without any statistically significant difference. Furthermore, the results showed no statistically significant differences in the patients with age <45 years (THW: 80.0%, rhTSH: 76.9%, *p* = 0.572), with age ≥45 years (THW: 80.8%, rhTSH: 76.4%, *p* = 0.271), of male sex (THW: 77.3%, rhTSH: 77.6%, *p* = 0.591), of female sex (THW: 81.7%, rhTSH: 77.4%, *p* = 0.238), with extrathyroidal extension (THW: 72.4%, rhTSH: 70.5%, *p* = 0.718), without extrathyroidal extension (THW: 85.7%, rhTSH: 81.7%, *p* = 0.296), without LN metastasis (THW: 86.6%, rhTSH: 84.3%, *p* = 0.532), with LN metastasis (THW: 70.5%, rhTSH: 66.2%, *p* = 0.456), with low‐risk staging (stages I–II, THW: 80.1%, rhTSH: 79.0%, *p* = 0.766), and with high‐risk staging (stages III–IV, THW: 81.8%, rhTSH: 69.8%, *p* = 0.074) (Table 2).

**TABLE 2:** Rate of excellent response with thyroid hormone withdrawal or recombinant human thyrotropin use among subgroups

		Excellent response (%)	*p*
All patients	THW (*n* = 323)	80.5	0.221
	rhTSH (*n* = 324)	76.5	
Age, years			
Age < 45	THW (*n* = 110)	80.0	0.572
	rhTSH (*n* = 108)	76.9	
Age ≥ 45	THW (*n* = 213)	80.8	0.271
	rhTSH (*n* = 216)	76.4	
Sex			
Male	THW (*n* = 88)	77.3	0.591
	rhTSH (*n* = 72)	73.6	
Female	THW (*n* = 235)	81.7	0.238
	rhTSH (*n* = 252)	77.4	
TNM stage (AJCC‐7th edition)			
Low risk (stage I + II)	THW (*n* = 246)	80.1	0.766
	rhTSH (*n* = 238)	79.0	
High risk (stage III + IV)	THW (*n* = 77)	81.8	0.074
	rhTSH (*n* = 86)	69.8	
Extrathyroidal extension			
Yes	THW (*n* = 127)	72.4	0.718
	rhTSH (*n* = 149)	70.5	
No	THW (*n* = 196)	85.7	0.296
	rhTSH (*n* = 175)	81.7	
Lymph node metastasis			
Yes	THW (*n* = 122)	70.5	0.456
	rhTSH (*n* = 139)	66.2	
No	THW (*n* = 201)	86.6	0.532
	rhTSH (*n* = 185)	84.3	

Abbreviations: AJCC, American Joint Cancer Committee; rhTSH, recombinant human thyrotropin; THW, Thyroid hormone withdrawal; TNM, tumor‐node‐metastasis.

**FIGURE 2:**
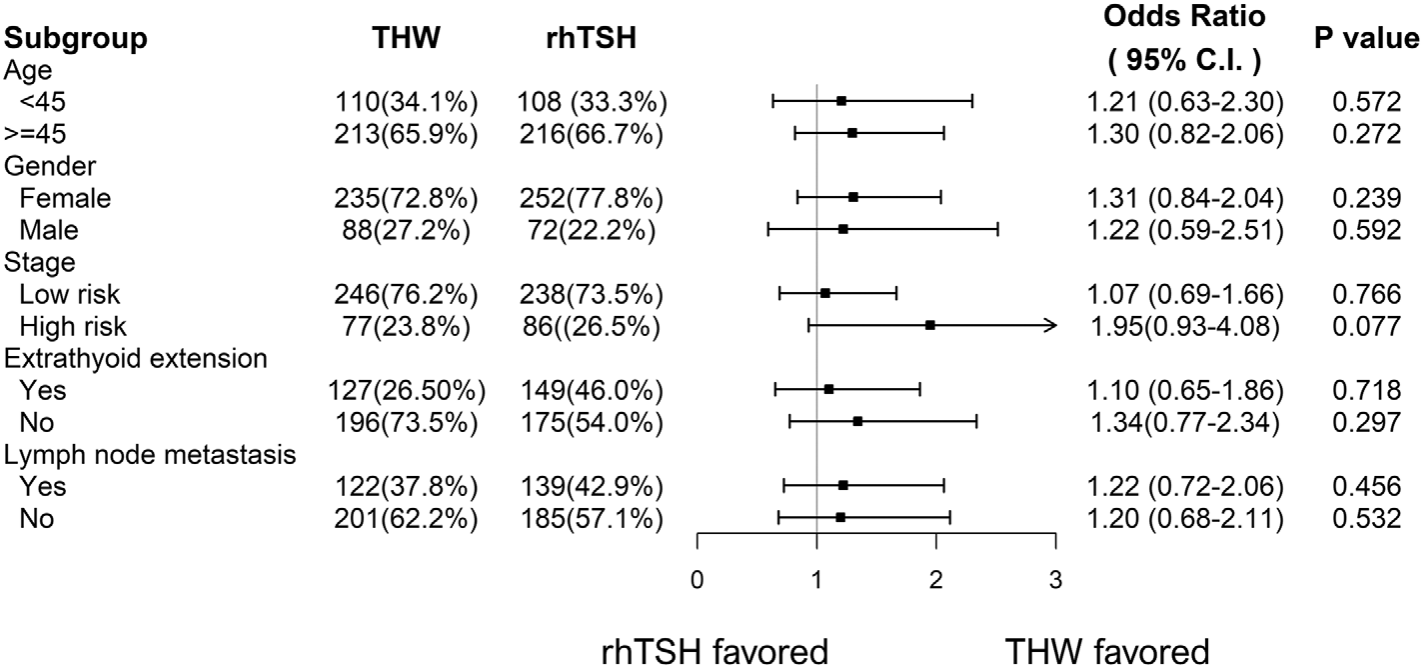
Comparison treatment response of THW versus rhTSH between different subgroups in PTC. Logistic regression analysis revealed no statically significant differences among different clinical characteristics stratified by age, sex, extrathyroidal extension, lymph node metastasis, and tumor stage. rhTSH, recombinant human thyrotropin; THW, thyroid hormone withdrawal; TNM, tumor‐node‐metastasis.

The logistic regression analysis was summarized in a forest plot, which also revealed no statistical significance in all the subgroups (Figure 2). Notably, high‐risk TNM staging showed favorable trends with THW, but the results were not statistically significant (*p* = 0.074) (Figure 2). Multivariate regression analyses adjusted the potential confounders in our study, which revealed extrathyroidal extension, LN metastasis, and high I^131^ dose as the prognostic factors affecting the excellent response rate in our study (Table 3).

**TABLE 3:** Multivariate analysis of baseline characteristics between patients with thyroid hormone withdrawal and those with recombinant human thyrotropin use

Characteristics	Odds ratio	(95% CI)	*p*
Age (years, ≥45 vs. <45)	1.18	0.74–1.88	0.484
Sex (male vs. female)	1.14	0.73–1.78	0.877
Stage (high risk vs. low risk)	1.51	0.90–2.54	0.122
Extrathyroidal extension	1.60	1.04–2.46	0.032
Lymph node metastasis	1.84	1.18–2.87	0.008
rhTSH use	1.17	0.79–1.74	0.430
I^131^ dose (mci)	1.01	1.01–1.02	<0.001

Abbreviations: CI, confidence interval; rhTSH, recombinant human thyrotropin.

## 4 DISCUSSION

In this study, the data demonstrated that there was no difference in the excellent response rate between the two methods after 1 year, irrespective of whether the patients received rhTSH or THW for the RIA preparation in Taiwan. Some previous large prospective studies have discussed the successful ablation rate as the primary outcome, and the majority focused on Western populations only.^10^, ^21^ However, there is limited evidence in Taiwan that compares the outcomes of THW and rhTSH for the pre‐RIA preparation by using a subgroup analysis.

Castagna et al. reviewed 512 patients with DTC, who were initially classified as low‐ to high‐risk patients following an operation, based on the pathology report and I^131^ uptake in a whole body scan after receiving RIA. The results revealed that the delayed risk stratification after an initial treatment of 8–12 months could predict the disease recurrence more accurately.^20^ Vaisman et al. reviewed 506 patients with DTC followed for a median of 10 years after total thyroidectomy and RIA in Brazil. The data validated the ATA dynamic risk stratification as an excellent initial predictor of recurrent/persistent disease.^17^ Although this study did not provide the long‐term survival outcome data, it still provided valuable clinical evidence to physicians in Taiwan.

Our observation results were similar to those of previous retrospective studies, which showed similar excellent response rates with rhTSH and THW as the RIA preparation in intermediate‐ or high‐risk recurrence patients.^10^, ^29^, ^30^ In the past, several large randomized trials investigating lower or higher radioiodine activity for ablation revealed similar clinical effects under THW or rhTSH injection for RIA ablation. Schlumberger et al.^10^ enrolled 684 patients in France for a randomized study with T1‐T2, NX‐N1, and M0. The ablation rate of rhTSH injection is equivalent to that of THW, both of which were used as the RIA preparation. Mallick et al.'s study, which included 29 centers in the United Kingdom and the patients had T1–T3 stage tumors and LN involvement, showed similar ablation rates between rhTSH and THW.^21^ Schlumberger et al. enrolled low‐risk DTC patients, and Mallick et al. enrolled high‐risk ones. Both prospective studies also compared the effect of low‐dose (1·1 GBq) or high‐dose (3·7 GBq) I^131^ for RIA in the rhTSH/THW groups. The ablation success rate was equivocal between rhTSH and THW for the RIA preparation, irrespective of the I^131^ dose (Table 4). For Asian population, a retrospective Korean study by Joung et al. on low‐ (30 mCi) and high‐dose (100 mCi) RIA ablation reported equivalent efficacy in the cases of rhTSH and THW before ablation.^30^ The results of Joung et al.'s study were consistent with those of Schlumberger et al.'s and Mallick et al.'s studies: rhTSH is as effective as THW in the preparation for RIA treatment.

**TABLE 4:** Comparison of the present study with previous studies

	Schlumberger et al.^10^	Mallick et al.^21^	Joung et al.^30^	Tsai et al.^31^	This study
Number of patients	684	421	570	88	647
Country	France	The United Kingdom	Korea	Taiwan	Taiwan
Design	Prospective	Prospective	Retrospective	Retrospective	Retrospective
Tumor stage	T1N0, T1N1, T1NX, T2N0, M0	T1–T3, N0–N1, M0	T1–T3, N0–N1, M0	M1 with any T, any N	T1–T3, NX‐N1, M0–M1
Criteria of successful ablation	Ultrasonography, sTg (≦1 ng/ml) and undetectable serum anti‐Tg antibody	Ultrasonography, sTg (<2 ng/ml) and undetectable serum anti‐Tg antibody	sTg <1 to 2 ng/ml, undetectable serum anti‐Tg antibody		Ultrasonography, Tg and anti‐Tg antibody
Outcome	rhTSH 91.7%, THW 92.9%	Ablation success rate: rhTSH 87.1%, THW 86.7%	Ablation success rate: 30 mCi: rhTSH 78.2%–80.5%, THW 71.8%–77% 100 mCi: rhTSH 71.8%–74.8%, THW 66.5%–74.8%	10‐year DSS rate: THW: 62.2% rhTSH: 73.3% 10‐year PFS rate: THW: 47.7% rhTSH: 71%	Excellent response rate THW: 80.5% rhTSH: 76.5%

Abbreviations: DSS, disease‐specific survival; PFS, progression‐free survival; rhTSH, recombinant human thyrotropin; Tg, thyroglobulin; THW, thyroid hormone withdrawal; TNM, tumor‐node‐metastasis; sTg, stimulated thyroglobulin.

To the best of our knowledge, this study is the first in Taiwan to retrospectively compare the treatment response rate of THW and rhTSH by using a subgroup analysis. The results of this study were consistent with those of previous studies (Table 4). Present evidence of Taiwan regarding rhTSH and THW for RIA preparation was presented by Tsai et al.: the long‐term survival rates in DTC patients with distant metastases revealed that rhTSH stimulation was safe and feasible in patients with distant metastases from PTC.^31^ However, our study population was more general, consisting of T1‐T3, NX‐N1, and M0‐M1. Tsai et al. did not analyze the effect of low or high I^131^ dosage, which was analyzed by previous studies.

Thus far, no sufficient data for Taiwan have been presented to compare the excellent response rate in low‐ to high‐risk DTC patients with rhTSH or THW as the pre‐RIA preparation. This study provides more evidence in Taiwan, which can help physicians and patients make decisions with a relatively strong support of evidence. However, this study has some limitations. First, this study had a retrospective design and lack of randomization, long‐term clinical outcome, and survival/recurrence rate. Second, the dosage of I^131^ was individualized and varied (30–200 mci) in this retrospective study. Third, the healthcare system in Taiwan does not offer rhTSH to patients who will receive RIA routinely, excluding THW intolerance or thyroid cancer recurrence. Most rhTSH users in Taiwan are also self‐paid, following shared decision making, which makes a *random* process impossible. The healthcare policy may lead to bias and/or increased rhTSH use in patients with a relatively large number of comorbidities. This reason may lead to a higher excellent response rate in high‐risk patients, although without any statistically significance. Fourth, this study did not consider long‐term clinical outcomes; therefore, more follow‐up time is required to collect sufficient data for a long‐term clinical outcome analysis.

In conclusion, our results indicated no prognostic implication differences by two different pre‐RIA preparations in DTC, even among different clinical characteristics stratified by age, sex, extrathyroidal extension, LN metastasis, and tumor stage.

## CONFLICTS OF INTEREST

All authors declare no conflict of interest.
